# Abrogation of transforming growth factor-β-induced tissue fibrosis in TBRI^ca^Col1a2Cre transgenic mice by the second generation tyrosine kinase inhibitor SKI-606 (Bosutinib)

**DOI:** 10.1371/journal.pone.0196559

**Published:** 2018-05-02

**Authors:** Peter J. Wermuth, Sergio A. Jimenez

**Affiliations:** Jefferson Institute of Molecular Medicine and Scleroderma Center, Thomas Jefferson University, Philadelphia, PA, United States of America; University of Bergen, NORWAY

## Abstract

Transforming growth factor-β (TGF-β) plays a crucial role in the pathogenesis of Systemic Sclerosis (SSc) and other fibrotic disorders. TGF-β-mediated c-Abl and Src kinase activation induces strong profibrotic cascade signaling. The purpose of this study was to test *in vivo* the antifibrotic activity of Bosutinib (SKI-606), a second generation c-Abl and Src kinase inhibitor, on TGF-β induced cutaneous and pulmonary fibrosis. For this purpose, we employed the TBRI^ca^Col1a2Cre transgenic mice expressing an inducible constitutively active TGF-β receptor 1 constitutively activated by Col1a promoter-mediated Cre recombinase. The mice were treated parenterally with 2.5, 5.0 or 10.0 mg/kg/day of Bosutinib for 42 days. Skin and lungs from control and Bosutinib-treated mice (n = 6 per group) were assessed by histopathology, measurement of tissue hydroxyproline content, PCR analysis of tissue fibrosis associated gene expression, and evidence of myofibroblast activation. Mice with constitutive TGF-β-1 signaling displayed severe cutaneous and pulmonary fibrosis. Bosutinib administration decreased collagen deposition and hydroxyproline content in the dermis and lungs in a dose-dependent manner. Bosutinib also reversed the marked increase in profibrotic and myofibroblast activation-associated gene expression. These results demonstrate that constitutive TGF-β-1-signaling-induced cutaneous and pulmonary fibrosis were abrogated in a dose-related manner following parenteral administration of the c-Abl and Src tyrosine kinase inhibitor, Bosutinib. These results indicate that Bosutinib may be a potential therapeutic agent for tissue fibrosis in SSc and other fibroproliferative disorders.

## Introduction

Fibrotic disorders including Systemic Sclerosis (SSc) are characterized by exaggerated local or systemic pathologic extracellular matrix (ECM) and fibrous tissue accumulation. These disorders are responsible for high morbidity and mortality worldwide [1–7]. Despite distinct and varied etiologies of fibrotic disorders and a remarkable heterogeneity in their clinical manifestations several common molecular mechanisms and signaling pathways regulate their pathogenesis [8–11]. A hallmark of these diseases is the increased expression, production and tissue accumulation of ECM proteins including the fibrillar types I, III and VI collagens and fibronectin, disrupting the normal architecture of the affected organs resulting in their dysfunction and eventual failure. The chronic and progressive nature of fibrotic diseases, the large array of affected organs, and the lack of effective disease modifying therapeutics render them a challenge to efficient medical management [1–3,8]. The mortality attributed to fibrotic diseases in Western countries may be as high as 45% or even higher in less developed and developing nations [1–3,8].

The fibroproliferative phenotype is largely mediated by activated myofibroblasts [12–15], the cells responsible for the increased production of various ECM macromolecules, including fibrillar type l, type lll, and type VI collagens and fibronectin, and a concomitant reduction of ECM-degrading matrix metalloproteinase activity. Activated myofibroblasts are characterized by the expression and production of α-smooth muscle actin (α-SMA), a protein that confers these cells a contractile phenotype allowing their migration and the extension of tissue damage. Although several cytokines and growth factors, such as transforming growth factor β (TGF-β), platelet-derived growth factor (PDGF), and connective tissue growth factor, play important roles in tissue fibrosis, the molecular pathways responsible are not completely understood [9,16–19].

SSc is a systemic autoimmune disease characterized by exaggerated and often progressive skin and multiple internal organ fibrosis leading to severe organ damage and high mortality [4,5]. Although its etiology is unknown, TGF-β signaling is crucial in SSc pathogenesis [5,9,17,18,20], rendering it an attractive target for SSc-disease-modifying therapies. In normal cells, TGF-β isoforms initiate signaling following binding to membrane-associated serine/threonine protein kinase TGF-β receptors [21]. The TGF-β type I and type II receptors (TBRI and TBRII, respectively) are present as homodimers in the membrane. Binding of the dimerized TGF-β ligand to the TBRII homodimer allows for the recruitment of the TBRI (also known as ALK5) homodimer and triggers the formation of a mature heterotetramer receptor complex, allowing the constitutively active TBRII kinase to autophosphorylate and then transphosphorylate the TBRI kinase [22]. Signaling by this activated receptor complex can then proceed through a canonical pathway or by one or more non-canonical pathways. In canonical TGF-β signaling, the C-terminal regions of the SMAD2 and SMAD3 proteins [23,24] are phosphorylated by the activated TBRI [25], allowing association with the co-mediator SMAD4 and nuclear accumulation of the SMAD2/3 complex where it can then regulate transcription of downstream genes [26]. Several non-canonical signaling pathways that do not depend on SMAD phosphorylation and nuclear accumulation have been described and include the mitogen activated protein kinases (MAPKs) ERK, p38 and JNK, PI3K/Akt and Rho GTPase pathways [27–29]. This diversity of signaling pathways involved mediate the pleiotropic downstream effects of TGF-β on cellular and molecular processes. Upregulation of TGF-β and its receptors [30–32] and of the TGF-β-regulated gene CCN2 or connective tissue growth factor (CTGF) also known as CCN2 has been reported in SSc fibroblasts and tissue samples [33], although the increased expression of CCN2/CTGF is Smad3-independent but Smad1-dependent [34,35]. Investigation of the role of TGF-β signaling in the induction of a fibrotic phenotype in SSc fibroblasts using small molecule inhibitors, antibodies, siRNA directed against the TGF-β receptors or SMADs as well as knockout mouse models has indicated the involvement and dysregulation of both canonical [36–38] and non-canonical [39–41] pathways.

Despite these studies implicating dysregulation of TGF-β signaling in mediating the induction of fibrosis in SSc, therapeutic approaches aimed at inhibition of TGF-β signaling have not been successful, largely owing to serious side effects caused by inhibition of the multiple pleiotropic effects of TGF-β as well as by crosstalk with numerous associated molecular signaling pathways. For example, metelimumab (CAT-192), a monoclonal antibody against TGF-β1, induced significant morbidity and mortality in a study of 45 patients with early-stage diffuse cutaneous SSc. CAT-192 also showed no evidence of efficacy compared to placebo as evaluated by changes in the modified Rodnan skin thickness score (MRSS), assessment of organ-based involvement and measurement of clinical biochemical parameters such as serum levels of soluble interleukin-2 receptor, collagen propeptides or of tissue levels of mRNA for procollagens I and III or for TGF-β1 [42]. A randomized, double-blind, multicenter, placebo-controlled trial utilizing CAT-152, a monoclonal antibody directed against TGF-β2, to evaluate its effect in preventing the progression of fibrosis in patients undergoing an initial trabeculectomy for primary open-angle or chronic angle-closure glaucoma demonstrated no statistically significant improvement compared to the placebo group over the course of the 12 month study [43]. In contrast, fresolimumab, an antibody that targets all three TGF-β isoforms improved clinical symptoms in a small proof-of-concept study of 15 SSc patients for 24 weeks [44]. Clinical improvement was measured by evaluating changes in the MRSS score as well as changes in the expression levels of the TGF-β-regulated biomarker genes thrombospondin-1 (THBS1) and cartilage oligomeric protein (COMP).

Modifying the activity of various tyrosine kinases to target tissue fibrosis has been intensely investigated [45–48]. Recently much interest has focused on the non-receptor Src kinases owing to their participation in various signal transduction pathways regulating important cellular processes including cell migration, apoptosis, cytoskeletal rearrangements, and cellular proliferation and differentiation [49–52]. Several profibrotic growth factors including PDGF and TGF-β activate Src kinase signaling by stimulating the phosphorylation of a tyrosine residue in its catalytic region [51,52]. Furthermore, several molecules involved in TGF-β-induced conversion of quiescent fibroblasts to activated myofibroblasts, such as focal adhesion kinase (FAK), hydrogen peroxide inducible gene 5 (HIC-5), myocardin-related transcription factor (MRTFA), and extracellular signal-regulated kinase 1/2 (ERK1), are regulated by Src and contribute to the persistent profibrotic phenotype of SSc fibroblasts [53–59]. Therefore, Src kinase inhibitors represent potentially novel and effective agents for treating fibrotic diseases.

Imatinib mesylate, a small-molecule tyrosine kinase inhibitor that targets Abl kinase activity associated with the Bcr-Abl translocation found in chronic myelogenous leukemia (CML) also blocks the activity of PDGFR, c-kit and c-fms (also known as colony stimulating factor 1 receptor or CSF1R). Treatment of SSc fibroblasts *in vitro* with imatinib inhibited the expression and production of several extracellular matrix components, including both type I collagen alpha 1 (Col1a1), and alpha 2 Col1a2 chains as well as fibronectin-1 in a dose-dependent manner and did not induce compensatory changes in the expression of matrix metalloproteinases or of tissue inhibitors of matrix metalloproteinases [60,61]. These effects were confirmed *in vivo* utilizing several animal models of systemic and tissue-specific fibrosis [60–64]. Furthermore, several small case series and case reports in SSc patients have shown improvement [65–67], and one study reported that imatinib induced improvement in two patients with Nephrogenic Systemic Fibrosis [68]. A randomized, placebo-controlled, double-blind trial examining the effect of imatinib treatment over 96 weeks in patients with idiopathic pulmonary fibrosis found imatinib treatment was well tolerated in these patients, although there was no effect on either survival or of clinical outcomes measures such as forced vital capacity, or other lung functions compared to the placebo group [69]. Second generation Abl inhibitors dasatinib and nilotinib have demonstrated similar anti-fibrotic effects in an animal model of fibrosis [70].

Bosutinib, or SKI-606 is a third-generation tyrosine kinase inhibitor developed to inhibit the BCR-Abl kinase responsible for Philadelphia chromosome positive CML. Bosutinib was approved for treating chronic-, accelerated-, and blast-phase CML in patients resistant to Imatinib [71,72]. Although BCR-Abl is not involved in tissue fibrosis pathogenesis, Bosutinib also inhibits the profibrotic c-Abl tyrosine kinase [73]. Bosutinib is also a potent Src kinase inhibitor, however, the kinase inhibitory effects of Bosutinib do not perturb the PDGF signaling pathway whose inhibition has been associated with severe side effects [74,75]. Indeed, Bosutinib therapy induces substantially less fluid retention and cardiac conduction problems compared with the first- and second-generation Src inhibitors Imatinib and Nilotinib, respectively [76].

The effects of Bosutinib on the expression and production of pro-fibrotic molecules and on the phenotypic transition of normal to activated myofibroblasts in dermal fibroblasts from SSc patients were examined previously *in vitro* [77]. The results showed a potent and dose-dependent inhibition of the increased gene expression of the profibrotic extracellular matrix proteins COL1A2, COL3A1, FN1 and of the profibrotic growth factor CTGF/CCN2 and a marked reduction of the increased production of the corresponding proteins in cultured dermal SSc fibroblasts. Bosutinib also abrogated the conversion of normal dermal fibroblasts into activated myofibroblasts as assessed by cellular levels of the myofibroblast marker α-SMA and reversed the abnormal profibrotic phenotype of dermal fibroblasts cultured from patients with diffuse SSc of recent onset [77]. Additionally, the highest concentration of Bosutinib examined (5 nM) mediated a 30% reduction in the amount of secreted collagen in SSc fibroblasts and a nearly 40% reduction in the amount of collagen and fibronectin in SSc fibroblast lysates. The studies described here are, to our knowledge, the first demonstration of potent antifibrotic effects of Bosutinib *in vivo* in a highly relevant transgenic mouse model of tissue fibrosis [78,79].

## Methods

### Experimental animals

All animal studies were reviewed and approved by the Institutional Animal Care and Use Committee at Thomas Jefferson University. Mice overexpressing a constitutively-activated fibroblast-specific form of the TGF-β receptor I under the control of the Col1α2 collagen gene promoter requiring tamoxifen for activation (TBRI^ca^ Col1a2-Cre) were employed in these studies. These mice develop extensive cutaneous and lung fibrosis following TBRI^ca^ expression in fibroblasts. TBRI^ca^ activation in these mice (provided by Dr. Benoit de Crumbrugghe) [78,79]was achieved by intraperitoneal injection of 1 mg 4-OH tamoxifen daily for 5 days. Only male mice were used given the remarkable gender differences in tissue fibrosis extent and severity [80,81]. Following the initial tamoxifen injection, mice were divided into 4 groups of 6 mice each and anesthetized with ketamine and xylazine followed by implantation of subdermal Alzet osmotic pumps (model #2006) containing either saline (control mice) or 2.5, 5.0 or 10.0 mg/kg/day Bosutinib dispensing their contents at a constant rate of 0.25 μl/hr for 42 days. Lidocaine was administered subcutaneously adjacent to the incision site pre- and post-operatively to minimize discomfort of the animals.

### Histopathological tissue analysis and determination of tissue hydroxyproline content

All mice were sacrificed at 42 days post pump implantation by CO_2_ asphyxiation and full thickness skin samples were excised from the dorsum of each mouse and both lungs were isolated. A portion of each tissue sample was fixed in 10% buffered formalin, embedded in paraffin, and sections (5 μM thickness) were obtained and stained with hematoxylin and eosin or with Masson’s trichrome.

A portion of the skin and lung samples isolated from each animal was weighed immediately following removal and acid-hydrolyzed overnight in 6N HCl at 107°C. The hydrolysates were assayed for their total hydroxyproline content as described [82,83]. The hydroxyproline content per mg wet tissue was determined by comparing the absorbance of each sample to a standard curve generated by assay of known amounts of 4-hydroxyproline. The hydroxyproline values were converted to amounts of collagen using a conversion factor of 7.5 since hydroxyproline represents ~13.5% of the amino acid content of collagen [82].

### RNA isolation and real-time polymerase chain reaction (RT-PCR)

Total RNA was extracted from skin and lung samples using Trizol and first-strand cDNA was generated using SuperScript II Reverse Transcriptase (Invitrogen). Transcript levels of genes encoding ECM macromolecules, myofibroblast differentiation and activation proteins, and downstream TGF-β transcription factors were determined using SYBR Green real-time PCR as previously described [84,85]. Primers were designed using Primer Quest (Integrated DNA Technologies) and were validated for specificity. The sequences of the primers employed are shown in **Table 1**. Differences in mRNA transcript levels in each PCR were corrected for 18S RNA endogenous control transcript levels; levels in control mice were set at 100% and all other values were expressed as multiples of the control values.

**Table 1:** Sequence of primers used for RT-PCR.

Gene	Forward Primer (5’-3’)	Reverse Primer (5’-3’)
18S	ACCAGAGCGGAAAGCATTTGCCA	TCGGCATCGTTTATGGTCGGAA
Acta2/ Sma	GACTCTCTTCCAGCCATCTTTC	GACAGGACGTTGTTAGCATAGA
Col1a1	GCATGGCCAAGAAGACATCG	TCCACGTCTCAGCATTGGG
Col3a1	AGCTTTGTGCAAAGTGGAACCTGG	CAAGGTGGCTGCATCCCAATTCAT
Comp	CGTGGGCTGGAAGGATAAA	TACTAGCTCAGGACCCTCATAG
Ctgf	ACTATGATGCGAGCCAACTG	CTCCAGTCTGCAGAAGGTATTG
Erk1	CTGGCTTTCTGACGGAGTATG	AGACCAGATGTCGATGGATTTG
Erk2	GTTGGTACAGAGCTCCAGAAA	GGAAGATAGGCCTGTTGGATAG
Fn1	TCCAGGACAACAGCATCAGTGTCA	CCACAGTGGGTTGCAAACCTTCAA
Fn-Eda	TAAAGGACTGGCATTCACTGA	GTGCAAGGCAACCACACTGAC
Hic5	GGAGGACCAATCTGAAGACAAG	TCAGTCTATCCAGTTCCTGAGT
Tgfb1	AAAGGCCACTGGGTAAAGGAGAGT	AAAGGCCACTGGGTAAAGGAGAGT
Tgfbr1	ATGTCCGCGTCCCACTA	CCAGAGTCTCTAGACTGTCCAT

### Statistical analyses

Data are expressed as mean ± standard deviation. Statistical significance of changes in gene expression levels or in hydroxyproline content was evaluated by Student’s t-test with a p value <0.05 deemed significant.

## Results

### Effects of Bosutinib on dermal fibrosis induced by constitutive TGF-β signaling in TBRI^ca^-Col1a2-Cre mice

**Fig 1:**
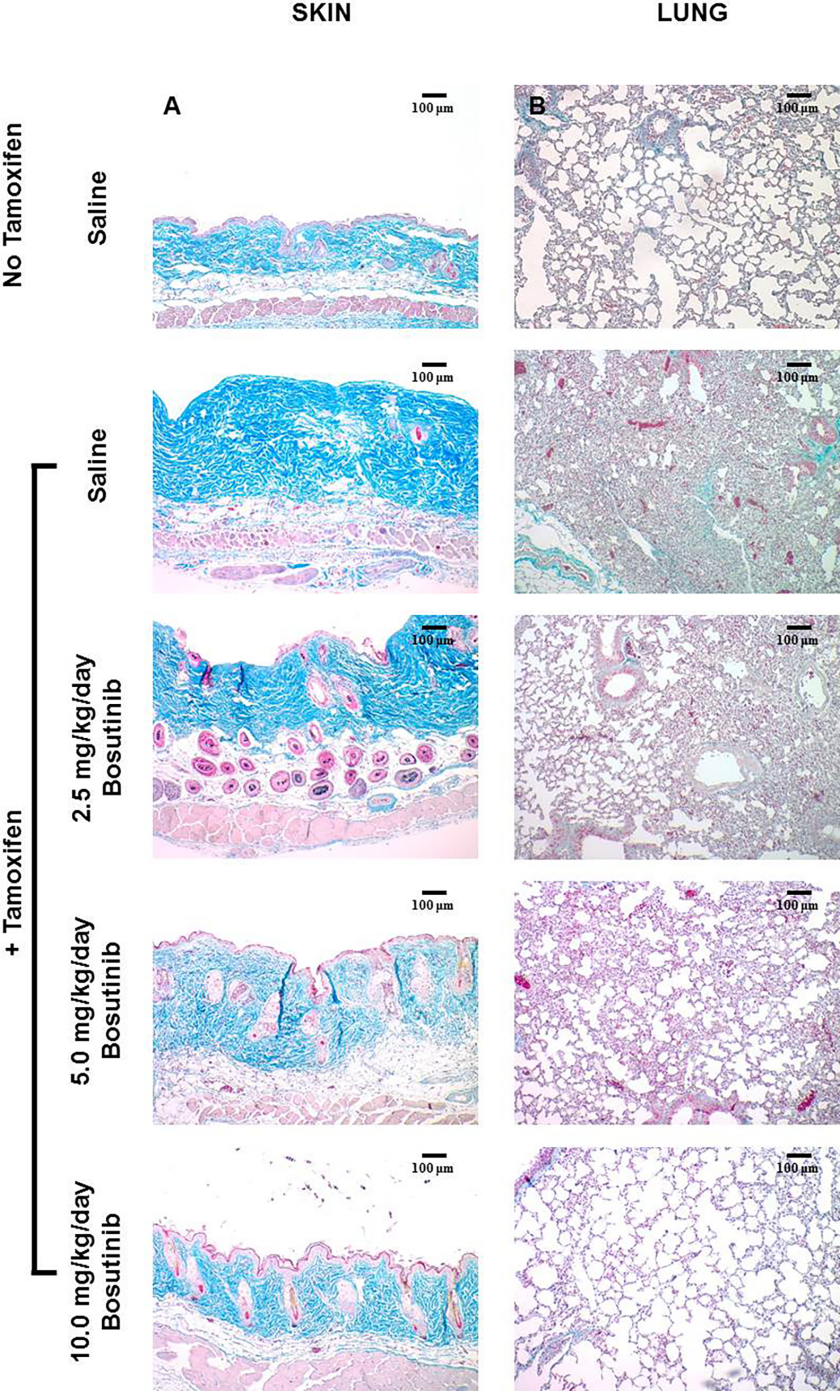
Histopathology of skin and lung from control, tamoxifen-injected untreated and tamoxifen-injected Bosutinib-treated TBRI^ca^-Col1a2-Cre transgenic mice. **A. Skin.** Skin sections from saline-treated tamoxifen-injected TBRI^ca^-Col1a2-Cre mice demonstrate increased dermal thickness and perivascular collagen accumulation (magnification 10X, **second left panel**) compared to skin sections from saline-treated non-tamoxifen-injected control mice (**upper left panel**). Skin sections from animals treated by subdermal osmotic pumps releasing 2.5 mg/kg/day, 5.0 mg/kg/day or 10 mg/kg/day of Bosutinib (**left panels**) display dose-dependent decreases in collagen deposition and dermal thickness compared with untreated mice. **B. Lung.** Sections from lung from saline-treated TBRI^ca^-Col1a2-Cre tamoxifen-injected mice (magnification 10X, **second left panel**) demonstrate marked loss of alveolar morphology with tissue consolidation and thickening of alveolar septae and perivascular and interstitial collagen accumulation compared to lung sections from saline-treated non-tamoxifen-injected control mice (**upper left panel**). Lung sections from animals treated by subdermal osmotic pumps releasing 2.5 mg/kg/day, 5.0 mg/kg/day or 10 mg/kg/day of Bosutinib (**left panels**) display a dose-dependent partial restoration of alveolar morphology and decreased thickening of alveolar septae and perivascular and interstitial collagen accumulation compared with the untreated mice.

The antifibrotic effects of Bosutinib *in vivo* were examined employing a highly relevant murine model of tissue fibrosis induced by TGF-β overexpression in fibroblasts [80,81]. These mice, carrying a floxed constitutively active TGF-β receptor (TBRI) allele activated by tamoxifen-induced Cre recombinase expression under control of the fibroblast-specific Col1A2 promoter develop extensive cutaneous and lung fibrosis [78,79]. The mice employed here may be a more pathophysiologically relevant tissue fibrosis model than the more widely employed bleomycin-induced fibrosis model [86,87]. Masson’s trichrome-stained full thickness skin sections from saline-injected control mice displayed normal tissue architecture and collagen staining (**Fig 1A**), whereas tamoxifen-activated TBRI^ca^-Col1a2-Cre mice skin demonstrated striking fibrosis with a marked increase in dermal thickness, accumulation of densely packed and irregularly arranged collagen bundles, and a marked increase in ECM collagen in the dermis and subdermal tissues (**Fig 1A**). The hypodermis also showed marked collagen infiltration and decreased overall adipose layer thickness. Abnormal collagen deposition occurred between individual muscle fibers of the panniculus carnosus in tamoxifen-injected animals compared with control mice. The epidermis did not differ between the control and tamoxifen-activated groups.

Tamoxifen-injected TBRI^ca^-Col1a2-Cre mice treated with Bosutinib dispensed continuously for 42 days employing subdermal osmotic pumps displayed a dose-dependent decrease in dermal fibrosis compared with saline control TBRI^ca^-Col1a2-Cre animals injected with tamoxifen (**Fig 1A**). Even in mice receiving only 2.5 mg/kg/day of Bosutinib dermal thickness and collagen bundle accumulation were noticeably decreased. These changes were more evident in mice treated with 5 mg/kg/day Bosutinib with the maximal effect observed in 10 mg/kg/day Bosutinib-treated mice that displayed near normal levels of collagen and ECM deposition in the dermis and essentially normal hypodermal tissue and subdermal muscle layers.

### Effects of Bosutinib on pulmonary fibrosis induced by constitutive TGF-β signaling in TBRI^ca^-Col1a2-Cre mice

Trichrome staining of the lungs from untreated tamoxifen-injected animals displayed severe alterations of the normal alveolar architecture with extensive areas of fibrosis causing alveolar septae thickening and fibrotic parenchymal consolidation, compared to mock-injected (no tamoxifen) control mice (**Fig 1B**). Marked interstitial, perivascular and peribronchiolar collagen accumulation was present (**Fig 1B**). In contrast, tamoxifen-injected Bosutinib-treated TBRI^ca^-Col1a2-Cre mice (**Fig 1B**) displayed a dose-dependent decrease in pulmonary fibrosis and alveolar/parenchymal abnormalities compared with animals injected with tamoxifen alone. Even in mice receiving only 2.5 mg/kg/day of Bosutinib lung tissue consolidation and collagen deposition were markedly decreased with substantially less alveolar architecture distortion. These improvements were more evident in 10 mg/kg/day Bosutinib-treated mice which displayed near normal levels of collagen and ECM deposition and essentially complete abrogation of tissue consolidation.

### Hydroxyproline content in tissues of TBRI^ca^-Col1a2-Cre mice

**Fig 2:**
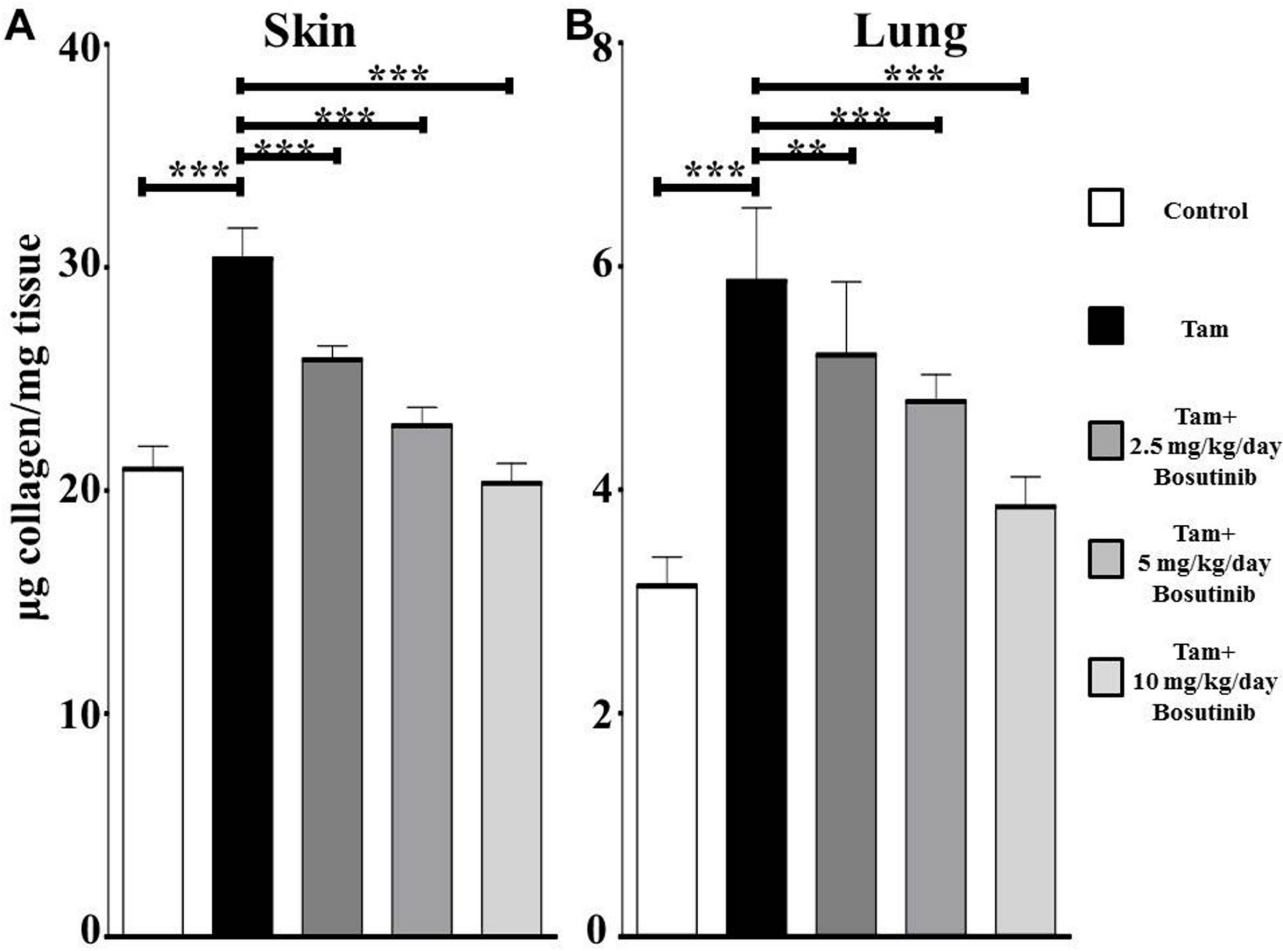
Hydroxyproline content of skin and lungs from control, tamoxifen-injected untreated and tamoxifen-injected Bosutinib-treated TBRI^ca^-Col1a2-Cre transgenic mice. A portion of skin (**A**) or lung (**B**) isolated from animals in each treatment group (n = 6) was hydrolyzed and analyzed for hydroxyproline content. The results were converted to total collagen tissue content and are expressed as μg/mg of tissue wet weight. The bars show the mean +/- standard error of each treatment group performed in triplicate. Significance determined by Student’s two-tailed t test. *: p<0.05, **: p<0.01, ***: p<0.001.

Hydroxyproline content of dorsal skin and lung samples was measured to assess quantitatively the extent of collagen deposition. Skin samples from tamoxifen-injected untreated mice contained approximately 1.5 fold greater hydroxyproline than control animals (**Fig 2A**). Bosutinib treatment resulted in a dose-dependent decrease in skin collagen content with collagen levels in skin from 10 mg/kg/day Bosutinib-treated mice not significantly different from the levels in control mice. Lung samples from tamoxifen-injected mice contained approximately 1.9 fold greater hydroxyproline than lungs from control mice (**Fig 2B**). Bosutinib treatment resulted in a dose-dependent decrease in lung collagen content with the collagen levels in lung from 10 mg/kg/day Bosutinib-treated mice slightly but not significantly elevated compared to the levels in control mice.

### Bosutinib abrogates the increased expression of genes encoding ECM macromolecules and myofibroblast differentiation proteins in tamoxifen-treated TBRI^ca^-Col1a2-Cre skin and lungs

**Fig 3:**
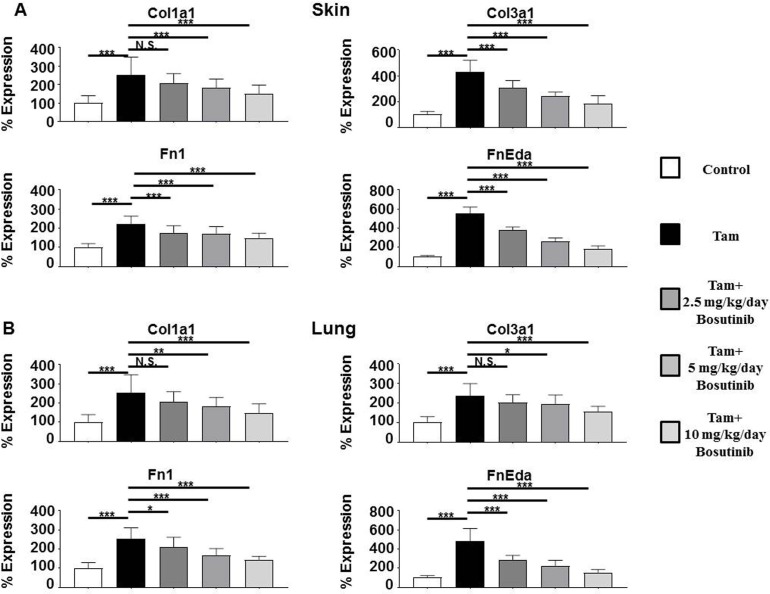
Expression of genes encoding ECM components in the skin and lungs from control, tamoxifen-injected untreated and tamoxifen-injected Bosutinib-treated TBRI^ca^-Col1a2-Cre transgenic mice. Expression of *Col1a1*, *Col3a1*, *Fn1*, and *Fn-Eda* in skin (**A**) and lung (**B**). The values shown are the mean (+/- SD) fold change levels of gene expression from each treatment group (n = 6) performed in triplicate for each tissue. Expression levels were normalized to 18S levels and the expression levels in untreated controls. Values for other samples are expressed relative to the normalized control. Significance was determined by Student’s T-test. Statistical significance: *: p<0.05, **: p<0.01, ***: p<0.001.

Changes in expression of genes encoding relevant ECM and profibrotic proteins in the skin and lungs of mice with constitutive TGF-β fibroblast signaling compared to saline-treated control mice were assessed by quantitative RT-PCR. Expression levels of the genes encoding *Col1a1* and *Col3a1* were upregulated in the skin by an average of 2.8 fold and 4.2 fold (**Fig 3A**), respectively and in the lungs by 2.5 fold and 2.4 fold, respectively, compared to tamoxifen-injected untreated mice (**Fig 3B**). *Fn1* expression was increased by 2.2 fold in the skin (**Fig 3A**) and by 4.8 fold in the lungs (**Fig 3B**) of tamoxifen-injected mice whereas the fibrosis-associated splice variant *Fn-Eda* displayed 5.5 fold and 2.5 fold increased expression in the skin and lung, respectively, compared to control animals. *Acta2/α-Sma* expression was increased 4 fold in the skin (**Fig 3A**) and 3.4 fold in the lung (**Fig 3B**) compared to saline injected animals whereas the levels of the growth factor *Ctgf/Ccn2* were increased 2.6 and 3.0 fold in the skin and lung, respectively. Bosutinib treatment produced a marked and dose-dependent decrease in the expression of all these genes in both skin and lung with the levels measured in 10 mg/kg/day Bosutinib-treated mice only slightly elevated compared to control untreated mice.

### Effect of Bosutinib on the increased Tgfb1 expression and of genes encoding TGF-β1 pathway components in tamoxifen-treated TBRI^ca^-Col1a2-Cre skin and lungs

**Fig 4:**
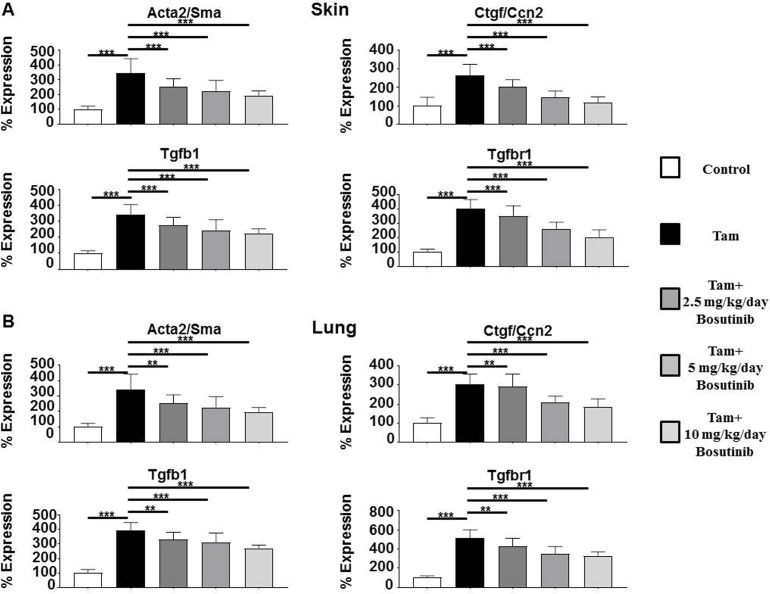
Expression of genes associated with myofibroblast transdifferentiation and with TGF-β signaling in the skin and lungs from control, tamoxifen-injected untreated and tamoxifen-injected Bosutinib-treated TBRI^ca^-Col1a2-Cre transgenic mice. Expression of *Acta2/Sma*, *Ctgf/Ccn2*, *Tgfb1*, and *Tgfbr1* in skin (**A**) and lung (**B**). The values shown are the mean (+/- SD) fold change levels of gene expression from each treatment group (n = 6) performed in triplicate for each tissue. Expression levels were normalized to 18S levels and the expression levels in untreated controls. Values for other samples are expressed relative to the normalized control. Significance was determined by Student’s T-test. Statistical significance: *: p<0.05, **: p<0.01, ***: p<0.001.

**Fig 5:**
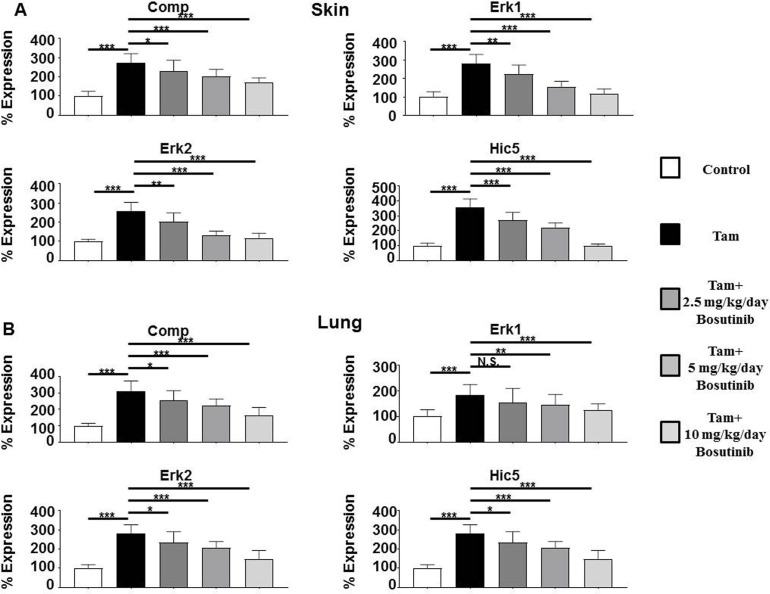
Expression of the TBF-β target genes *Comp* and *Erk1*, *Erk2* and the TGF-β-inducible *Hic5* gene in the skin and lungs from control, tamoxifen-injected untreated and tamoxifen-injected Bosutinib-treated TBRI^ca^-Col1a2-Cre transgenic mice. Expression of *Comp*, *Erk1*, *Erk2*, and *Hic5* in skin (**A**) and lung (**B**). The values shown are the mean (+/- SD) fold change levels of gene expression from each treatment group (n = 6) performed in triplicate for each tissue. Expression levels were normalized to 18S levels and the expression levels in untreated controls. Values for other samples are expressed relative to the normalized control. Significance was determined by Student’s T-test. Statistical significance: *: p<0.05, **: p<0.01, ***: p<0.001.

Profibrotic growth factor *Tgfb1* expression levels were upregulated by 3.4 fold in the skin and by 3.9 fold in the lungs of tamoxifen-injected animals compared to control animals and expression of its receptor *Tgfbr1* was upregulated 4 fold in the skin (**Fig 4A**) and 5.2 fold in the lung (**Fig 4B**), reflecting tamoxifen-induced transgene expression and the subsequent paracrine upregulation of TGF-β1 expression. Expression of the TGF-β-induced gene *Comp* was upregulated in the skin and lungs of tamoxifen-injected mice by 2.7 fold and 3.0 fold respectively (**Fig 5A and 5B**) and these increases were abrogated by Bosutinib. Bosutinib treatment of the mice induced a dose-dependent decrease in expression of these genes however, even at the 10 mg/kg/day dose, their expression remained significantly elevated compared to control untreated mice.

### Bosutinib abrogates the increased expression of genes encoding Erk1/2 protein kinases and Hic5

Expression levels of the TGF-β- and Src kinase-regulated *Erk1* and *Erk2* and of the TGF-β-induced *Hic5* gene were also examined. *Erk1* levels were upregulated by 2.8 fold in the skin and by 1.8 fold in the lungs of tamoxifen-injected mice compared to control mice and expression of the *Erk2* kinase was upregulated 2.6 fold and 2.8 fold in the skin and lungs, respectively. *Hic5* expression levels increased 3.6 fold in the skin and 2.6 fold in the lungs of tamoxifen-injected mice (**Fig 5A and 5B**). Bosutinib treatment induced a dose-dependent decrease in expression of these genes, and these levels were not significantly different in the 10 mg/kg/day treated mice from those measured in the skin and lungs of saline-treated control animals.

## Discussion

The effect of Bosutinib on the expression of genes encoding ECM components, regulation of myofibroblast transdifferentiation and TGF-β1 production was previously examined *in vitro* in SSc dermal fibroblasts [77]. Bosutinib induced a potent and dose-related inhibition of COL1A2, COL3A1, FN1 and CTGF expression in SSc dermal fibroblasts and potent inhibition of total collagen production by these cells. Intriguingly, the effect of Bosutinib on total collagen production was highly selective for SSc dermal fibroblasts since only minimal effects on normal fibroblasts were observed. Importantly, Bosutinib also mediated a significant reduction in the levels of α-SMA, a marker of myofibroblast activation of SSc fibroblasts [77]. The results of these *in vitro* studies suggested that Bosutinib may represent a novel, selective and effective antifibrotic agent for SSc therapy. The present study was performed to further validate this suggestion in an *in vivo* animal model of tissue fibrosis. Bosutinib was tested *in vivo* employing a highly relevant murine model of tissue fibrosis that possesses a fibroblast-specific tamoxifen-inducible constitutively active TGF-β receptor (TBRI) under control of the fibroblast-specific Col1A2 gene [78,79]. This model reproduces more accurately the effects of upregulated TGF-β signaling that has been associated with the establishment of a persistent profibrotic phenotype in SSc [86,87].

Following tamoxifen-induced transgene expression activation, three doses (2.5, 5.0 or 10.0 mg/kg/day) of Bosutinib were administered to the TBRI^ca-^Col1a2-Cre mice employing subdermal osmotic pumps that provide continuous release of Bosutinib over 42 days. Bosutinib effects on the development of tissue fibrosis in skin and lungs were assessed by: 1) immunohistochemistry; 2) measurement of tissue collagen content; 3) changes in the expression of genes encoding various ECM components; and 4) assessment of the transdifferentiation of fibroblasts to profibrotic activated myofibroblasts. Bosutinib administration resulted in a marked and dose-dependent decrease in skin and lung tissue fibrosis assessed by histopathologic analysis using Masson’s trichrome stain. In the skin, constitutive TGF-β signaling induced the expected increase in collagen deposition and dermal thickness whereas Bosutinib-treated animals displayed decreased collagen deposition and dermal thicknesses compared with the saline-treated animals. In the lung, constitutive TGF-β signaling induced dramatic tissue consolidation and alveolar thickening with abnormal and exaggerated perivascular and interstitial collagen deposition, whereas in Bosutinib-treated mice a striking restoration or improvement of tissue architecture and decreased collagen deposition was noted. These observations were confirmed by the analysis of collagen deposition in the skin and lungs by measurement of hydroxyproline levels. In both skin and lung, Bosutinib induced dose-dependent decreases in hydroxyproline levels compared to control non-Bosutinib treated animals.

An analysis of profibrotic gene expression levels in response to Bosutinib revealed its effect on multiple genes involved in the activation of myofibroblasts and encoding components of the extracellular matrix that are regulated by TGF-β signaling. *Col1a1*, *Col3a1*, and *Fn1* gene expression increased following activation of TGF-β signaling in fibroblasts as did the expression of the fibrosis-specific splice variant *Fn-Eda*. Bosutinib decreased expression of these genes in a dose-dependent manner, returning their expression levels to near the levels measured in non-tamoxifen control animals. A similar effect was observed on *Acta2/Sma* gene, and on expression of genes encoding the TGF-β-induced proteins *Comp* and *Hic5* and of the TGF-β-regulated growth factor *Ctgf/Ccn2*. Expression of *Tgfbr1*, the receptor encoded by the transgene was upregulated in tamoxifen-treated mice as was expression of *Tgfb1*. Bosutinib reduced expression of these genes in a dose-dependent manner although they remained elevated even at the 10 mg/kg/day dose most likely owing to the high level of transgene expression. The ability of Bosutinib to modify the TGF-β-mediated autocrine increased levels of TGF-β and of the TGF-β receptors may represent one mechanism for the antifibrotic effects that we observed in this model, however, since the levels of these genes remain upregulated compared to the levels measured in control animals, the available evidence indicates that the effects of Bosutinib on TGF-β-mediated skin and lung fibrosis are due to its suppression of the ability of Src/c-Abl kinases to mediate the downstream effects of TGF-β signaling. Finally, the expression levels of the genes encoding Erk1 and Erk2 kinases that were significantly upregulated following activation of constitutive TGF-β signaling were reduced in a dose-dependent manner following Bosutinib treatment.

In conclusion, Bosutinib displays potent anti-fibrotic effects in an *in vivo* model of TGF-β-mediated tissue fibrosis, reducing the development of fibrosis in the skin and lungs of treated animals in a dose-dependent manner as assessed by histopathologic analysis of collagen deposition, by measurement of the levels of hydroxyproline as a biochemical analysis of total collagen content in the isolated skin and lungs of these animals, and by the analysis of the expression of genes encoding ECM components, markers of myofibroblast activation and differentiation, and of relevant profibrotic kinases and other TGF-β1 molecular targets. The results obtained therefore extend the observations of the previously reported *in vitro* study of the antifibrotic effects of Bosutinib in SSc dermal fibroblasts [40] to an *in* vivo transgenic mouse model of TGF-β-mediated tissue fibrosis and overall reinforce the previously stated conclusion that Bosutinib [77] may represent a novel, selective and effective antifibrotic agent for therapy of SSc and other fibrotic disorders.

## Abbreviations

α-SMA: α-smooth muscle actin
BCR-Abl: B cell receptor-Abelson kinase
c-Abl: cellular-Abelson kinase
CML: chronic myeloid leukemia
Col1: Collagen, type I
Col3: Collagen, type 3
Comp: Cartilage oligomeric matrix protein
CTGF/CCN2: Connective tissue growth factor/Cysteine-rich 61, CTGF, Nephroblastoma-overexpressed 2
Cre: Cre recombinase
ECM: Extracellular matrix
ERK1/2: Extracellular signal-regulated kinase 1/2
FAK: Focal adhesion kinase
Fn1: Fibronectin 1
Fn-Eda: Fibronectin-Eda
Hic-5: Hydrogen peroxide inducible gene 5
KIT: c-kit tyrosine kinase
Mrtfa/Mkl1: myocardin-related transcription factor a/megakaryoblastic leukemia (translocation) 1
PDGF: Platelet derived growth factor
Src: cellular tyrosine kinase
SSc: Systemic sclerosis
TBRI^ca^: TGF-β receptor 1^constitutively activated^
TGF-β/tgfb: Transforming growth factor β
TBRI: TGF-β receptor 1
TBRII: TGF-β receptor 2
